# In-hospital mortality associated with community-acquired pneumonia due to methicillin-resistant *Staphylococcus aureus*: a matched-pair cohort study

**DOI:** 10.1186/s12890-021-01713-1

**Published:** 2021-11-03

**Authors:** Yukiyo Sakamoto, Yasuhiro Yamauchi, Taisuke Jo, Nobuaki Michihata, Wakae Hasegawa, Hideyuki Takeshima, Hiroki Matsui, Kiyohide Fushimi, Hideo Yasunaga, Takahide Nagase

**Affiliations:** 1 Department of Respiratory Medicine, Graduate School of Medicine, The University of Tokyo, 7-3-1, Hongo, Bunkyo-ku, Tokyo, 113-8655 Japan; 2 Department of Health Services Research, Graduate School of Medicine, The University of Tokyo, 7-3-1, Hongo, Bunkyo-ku, Tokyo, 113-8655 Japan; 3 Department of Respiratory Medicine, NTT Medical Center Tokyo, 5-9-22, Higashi-Gotanda, Shinagawa-ku, Tokyo, 141-8625 Japan; 4 Department of Clinical Epidemiology and Health Economics, School of Public Health, The University of Tokyo, 7-3-1, Hongo, Bunkyo-ku, Tokyo, 113-8655 Japan; 5 Department of Health Policy and Informatics, Tokyo Medical and Dental University Graduate School of Medicine, Tokyo, 1-5-45 Yushima, Bunkyo-ku, Tokyo, 113-8510 Japan

**Keywords:** Methicillin-resistant *Staphylococcus aureus* pneumonia, Community-acquired pneumonia, In-hospital mortality, Healthcare costs during hospitalisation

## Abstract

**Background:**

It remains unclear whether methicillin-resistant *Staphylococcus aureus* (MRSA) pneumonia is associated with higher mortality compared with non-MRSA pneumonia. This study’s objective was to compare outcomes including in-hospital mortality and healthcare costs during hospitalisation between patients with MRSA pneumonia and those with non-MRSA pneumonia.

**Methods:**

Using a national inpatient database in Japan, we conducted a 1:4 matched-pair cohort study of inpatients with community-acquired pneumonia from 1 April 2012 to 31 March 2014. In-hospital outcomes (mortality, length of stay and healthcare costs during hospitalisation) were compared between patients with and without MRSA infection. We performed multiple imputation using chained equations followed by multivariable regression analyses fitted with generalised estimating equations to account for clustering within matched pairs. All-cause in-hospital mortality and healthcare costs during hospitalisation were compared for pneumonia patients with and without MRSA infection.

**Results:**

Of 450,317 inpatients with community-acquired pneumonia, 3102 patients with MRSA pneumonia were matched with 12,320 patients with non-MRSA pneumonia. The MRSA pneumonia patients had higher mortality, longer hospital stays and higher costs. Multivariable logistic regression analysis revealed that MRSA pneumonia was significantly associated with higher in-hospital mortality compared with non-MRSA pneumonia (adjusted odds ratio = 1.94; 95% confidence interval: 1.72–2.18; *p* < 0.001). Healthcare costs during hospitalisation were significantly higher for patients with MRSA pneumonia than for those with non-MRSA pneumonia (difference = USD 8502; 95% confidence interval: USD 7959–9045; *p* < 0.001).

**Conclusions:**

MRSA infection was associated with higher in-hospital mortality and higher healthcare costs during hospitalisation, suggesting that preventing MRSA pneumonia is essential.

**Supplementary Information:**

The online version contains supplementary material available at 10.1186/s12890-021-01713-1.

## Introduction

Methicillin-resistant *Staphylococcus aureus* (MRSA) is a drug-resistant bacterium. The World Health Organization reported that MRSA infections accounted for 51.3% and 53% of *Staphylococcus aureus* infections in the United States and Japan, respectively [1].

Mortality from pneumonia caused by multidrug-resistant pathogens has been reported to be higher than that from pneumonia caused by other pathogens [2]; however, it remains unclear whether mortality from MRSA pneumonia is higher than mortality from non-MRSA pneumonia. Only a few studies have compared mortality between MRSA pneumonia and non-MRSA pneumonia; one showed a trend toward higher in-hospital mortality among MRSA pneumonia patients [3], whereas another showed that MRSA infection did not affect intensive care unit (ICU) mortality or in-hospital mortality in patients with ventilator-associated pneumonia [4]. These previous studies did not adjust for pulmonary comorbidities, which have been reported to be associated with in-hospital mortality in hospitalised patients with pneumonia [5].

The aim of the present study was to use a national inpatient database to examine the differences in in-hospital mortality and healthcare costs during hospitalisation between patients with MRSA pneumonia and those with non-MRSA pneumonia, adjusting for pulmonary comorbidities.

## Methods

### Data source

We used the Diagnosis Procedure Combination database, a nationwide inpatient administrative claims and discharge abstract database in Japan. This database contains data on main diagnoses, primary diagnosis, and comorbidities at admission, recorded using *International Classification of Diseases and Related Health Problems, 10th Revision* (ICD-10) codes and Japanese text data; age; sex; body height and weight; grade of activities of daily life on admission (Barthel Index) [6]; level of dyspnoea based on the Hugh-Jones classification [7]; ambulance use and discharge status. The database also includes the Age, Dehydration, Respiratory Failure, Orientation Disturbance and Blood Pressure (A-DROP) score for patients with community-acquired pneumonia, as well as data on whether C-reactive protein (CRP) was ≥ 20 mg/dL or infiltration covering at least two-thirds of one lung on chest radiography, mechanical ventilation during hospitalisation and hospitalisation costs. The sensitivity and specificity of the recorded ICD-10 codes and procedures in the Diagnosis Procedure Combination database were validated in a previous study [8].

The Hugh-Jones classification is a widely used dyspnoea scale with the following categories: I (the patient’s breathing is as good as that of other people of own age and build while working, walking and climbing hills or stairs), II (the patient is able to walk at the pace of normal people of the same age and build on level ground but is unable to keep up on hills or stairs), III (the patient is unable to keep up with normal people on level ground but is able to walk about a mile or more at their own pace), IV (the patient is unable to walk more than about 50 yards on level ground without resting), V (the patient is short of breath when talking or undressing or is unable to leave their home because of shortness of breath) and unspecified (the patient cannot be classified into any of the above grades because of bedridden status) [7].

The A-DROP score, established by the Japanese Respiratory Society, is a modified version of the CURB-65 (Confusion, Urea, Respiratory rate, Blood pressure-65) score [9]. The A-DROP score includes the following parameters: age (men: ≥ 70 years, women: ≥ 75 years), dehydration (blood urea nitrogen ≥ 21 mg/dL), respiratory failure (SaO_2_ ≤ 90% or PaO_2_ ≤ 60 mmHg), orientation disturbance (confusion) and low blood pressure (systolic blood pressure ≤ 90 mmHg).

The Institutional Review Board of The University of Tokyo approved this study and waived the requirement for patient informed consent because of the anonymous nature of the data.

### Patient selection

We retrospectively collected data on patients admitted to hospitals for community-acquired pneumonia who were discharged from 1 April 2012 to 31 March 2014. Community-acquired pneumonia was defined according to the 2019 guidelines on the management of adults with community-acquired pneumonia published by the Infectious Diseases Society of America/American Thoracic Society [10].

We defined MRSA pneumonia patients as those who had both the ICD-10 code for MRSA pneumonia and records of the administration of anti-MRSA antibiotics (vancomycin, linezolid, teicoplanin or arbekacin) for more than 7 days.

### Outcomes

The primary outcome of this study was all-cause in-hospital mortality. The secondary outcomes were 30-day in-hospital mortality, 90-day in-hospital mortality, length of stay and hospitalisation costs. The duration of antibiotic therapy was also evaluated.

### Statistical analysis

The χ^2^ test was used to compare proportions between groups. The two-sample *t*-test was used to compare average values, and the Mann–Whitney test was used to compare the median values between groups.

Among the patients with pneumonia, we selected an MRSA pneumonia group and a non-MRSA pneumonia group with 1:4 matching: for each patient in the MRSA pneumonia group, we identified four non-MRSA patients of the same sex who were admitted to the same hospital in the same year and whose ages were within 5 years of the age of the MRSA patient. We used hospital identifiers for matching to cancel out site-specific effects such as physician practice patterns and treatment outcomes [11].

We performed multiple imputation for missing data on body mass index (BMI), Barthel Index, Hugh-Jones grade, A-DROP score, CRP ≥ 20 mg/mL or infiltration covering at least two-thirds of one lung on chest radiography, and hospitalisation costs. We replaced each missing value with a set of substituted plausible values by generating 20 complete datasets using the multivariate imputation by chained equations method. The following covariates were used to create these 20 complete datasets: MRSA pneumonia, age, sex, fiscal year, haemodialysis, mechanical ventilation at admission, ICU admission, arrival by ambulance, chronic obstructive pulmonary disease (COPD), interstitial lung disease, aspiration pneumonia, *Pseudomonas aeruginosa* pneumonia, cerebrovascular disease, Parkinson disease, diabetes, dementia, in-hospital death, 30-day in-hospital death and 90-day in-hospital death, with the assumption that data were missing at random [12, 13]. Estimates from these 20 imputed datasets were combined using Rubin’s rule to obtain combined imputation estimates and standard errors.

Then, using multivariable logistic regression analysis fitted with generalised estimating equations to account for the 1:4 matched-pair clustering, we examined the factors associated with all-cause in-hospital mortality. Multiple linear regression analysis fitted with generalised estimating equations was also used to assess hospitalisation costs. The following independent variables were included in the models: age, sex, MRSA pneumonia, BMI, Barthel Index, Hugh-Jones grade, A-DROP score, CRP ≥ 20 mg/mL or infiltration covering at least two-thirds of one lung on chest radiography, haemodialysis, mechanical ventilation at admission, ICU admission, arrival by ambulance, COPD, interstitial lung disease, aspiration pneumonia and *Pseudomonas aeruginosa* pneumonia. For sensitivity analyses, we added independent variables of chronic heart failure, chronic liver disease, sepsis, acute renal failure, leukopenia, immunosuppression and stroke to the multivariable regression models used in the main analyses. Statistical analyses were performed using SPSS, Version 22.0 (IBM SPSS, Armonk, NY, USA) and Stata, Version 16 (StataCorp, College Station, TX, USA).

### Patient and public involvement

Patients or the public were not involved in the design, or conduct, or reporting, or dissemination plans of our research.

## Results

A total of 450,317 patients were hospitalised for community-acquired pneumonia during the study period, including 3102 patients with MRSA pneumonia. There were 44,854 in-hospital deaths (10.0%). The patients with MRSA pneumonia were matched with 12,320 patients hospitalised for non-MRSA pneumonia. The mean age was 79.4 years (standard deviation [SD] = 10.8), and 66.4% of the patients were male. Table 1 shows the characteristics of patients with MRSA pneumonia and non-MRSA pneumonia after 1:4 matching. The MRSA pneumonia group tended to have lower BMIs, lower activities of daily living scores, higher Hugh-Jones grades, higher A-DROP scores, CRP ≥ 20 mg/mL or infiltration covering at least two-thirds of one lung on chest radiography, mechanical ventilation on the day of admission or the following day, ICU admission on the day of admission or the following day, ambulance use, aspiration pneumonia and end-stage renal disease with haemodialysis. The percentages of patients with smoking history and COPD were lower in the MRSA pneumonia group than in the non-MRSA pneumonia group.

**Table 1:** Characteristics of patients with MRSA pneumonia and patients with non-MRSA pneumonia after 1:4 matching

	MRSA pneumonia		Non-MRSA pneumonia		*p*-value
	*n* (%)		*n* (%)		
Sex					0.943
Male	2059	(66.4)	8186	(66.4)	
Female	1043	(33.6)	4134	(33.6)	
Age (years, mean ± SD)	79.6 ± 10.7		79.5 ± 10.5		0.698
Body mass index (kg/m^2^)					< 0.001
< 18.5	1388	(44.7)	3539	(28.7)	
18.5–24.9	1136	(36.6)	5899	(47.9)	
25–29.9	116	(3.7)	1147	(9.3)	
≥ 30	28	(0.9)	199	(1.6)	
Missing	434	(14.0)	1536	(12.5)	
ADL score (Barthel Index)					< 0.001
85–100	474	(15.3)	4144	(33.4)	
60–80	159	(5.1)	991	(8.0)	
0–55	2024	(65.2)	5503	(44.7)	
Missing	445	(14.3)	1712	(13.9)	
Smoking history					0.003
Yes	1226	(39.5)	5239	(42.5)	
No	1876	(60.5)	7086	(57.5)	
Hugh-Jones grade					< 0.001
I	132	(4.3)	1604	(13.0)	
II	193	(6.2)	1774	(14.4)	
III	231	(7.5)	1411	(11.5)	
IV	435	(14.0)	2444	(19.8)	
V	948	(30.6)	2375	(19.3)	
Missing	1163	(37.5)	2712	(22.0)	
A-DROP score					< 0.001
0	162	(5.2)	1089	(8.8)	
1	639	(20.6)	3882	(31.5)	
2	870	(28.0)	3871	(31.4)	
3	701	(22.6)	2099	(17.0)	
4	410	(13.2)	791	(6.4)	
5	218	(7.0)	316	(2.6)	
Missing	102	(3.3)	272	(2.2)	
Dehydration					< 0.001
Yes	1788	(55.6)	5443	(44.2)	
No	1288	(41.5)	6779	(55.0)	
Missing	26	(0.8)	98	(0.8)	
Respiratory failure					< 0.001
Yes	1612	(52.0)	4591	(37.3)	
No	1468	(47.3)	7642	(62.0)	
Missing	22	(0.7)	87	(0.7)	
Orientation disturbance					< 0.001
Yes	941	(30.3)	1934	(15.7)	
No	2100	(67.7)	10,285	(83.5)	
Missing	61	(2.0)	101	(0.8)	
Systolic blood pressure					< 0.001
< 90 mmHg	502	(16.2)	1013	(8.2)	
≥ 90 mmHg	2581	(83.2)	11,243	(91.3)	
Missing	19	(0.6)	64	(0.5)	
CRP ≥ 20 mg/mL or infiltration covering at least two-thirds of one lung on chest radiography					< 0.001
Yes	477	(15.4)	1369	(11.1)	
No	1030	(33.2)	5185	(42.1)	
Missing	1595	(51.4)	5766	(46.8)	
Mechanical ventilation at admission					< 0.001
Yes	263	(8.5)	408	(3.3)	
No	2839	(91.5)	11,912	(96.7)	
ICU admission					< 0.001
Yes	101	(3.3)	157	(1.3)	
No	3001	(96.7)	12,163	(98.7)	
Haemodialysis					< 0.001
Yes	115	(3.7)	216	(1.8)	
No	2987	(96.3)	12,104	(98.2)	
Emergency transport					< 0.001
Yes	1061	(34.2)	3524	(28.6)	
No	2401	(65.8)	8796	(71.4)	
COPD					0.003
Yes	328	(10.6)	1548	(12.6)	
No	2774	(89.4)	10,772	(87.4)	
Interstitial lung disease					0.231
Yes	88	(2.8)	404	(3.3)	
No	3014	(97.2)	11,916	(96.7)	
Aspiration pneumonia					< 0.001
Yes	229	(7.4)	206	(1.7)	
No	2873	(92.6)	12,114	(98.3)	
Pseudomonas aeruginosa					0.635
Pneumonia					
Yes	67	(2.2)	287	(2.3)	
No	3035	(97.8)	12,033	(97.7)	
Diabetes					0.999
Yes	571	(18.4)	2268	(18.4)	
No	2531	(81.6)	10,052	(81.6)	

MRSA, methicillin-resistant *Staphylococcus aureus*; ADL, activities of daily living; A-DROP, Age, Dehydration, Respiratory Failure, Orientation Disturbance and Blood Pressure; CRP, C-reactive protein; ICU, intensive care unit; COPD; chronic obstructive pulmonary disease

All-cause in-hospital mortality was 31.2% in the MRSA pneumonia group, whereas it was 11.6% in the non-MRSA pneumonia group (Table 2). All-cause 30-day and 90-day mortality were also higher in the MRSA pneumonia group than in the non-MRSA pneumonia group. The duration of antibiotic therapy was longer in the MRSA pneumonia group than in the non-MRSA pneumonia group. Length of hospital stay was longer and hospitalisation costs were higher in the MRSA pneumonia group than in the non-MRSA pneumonia group.

**Table 2:** Outcomes of patients with MRSA pneumonia and patients with non-MRSA pneumonia after 1:4 matching

	MRSA pneumonia		Non-MRSA pneumonia		*p*-value
In-hospital mortality	967	(31.2)	1429	(11.6)	< 0.001
30-day mortality	382	(12.3)	1011	(8.20)	< 0.001
90-day mortality	809	(26.1)	1330	(10.8)	< 0.001
Length of stay (days)	35.0	[22–62]	14.0	[9–23]	< 0.001
Antibiotic therapy (days)	24.0	[15–45]	9.0	[6–13]	< 0.001
Hospitalisation costs (USD)	12,156	[7827–20,615]	4665	[3163–7298]	< 0.001

Data are expressed as numbers (%) or as medians [interquartile ranges]

MRSA, methicillin-resistant *Staphylococcus aureus*; USD, United States Dollar

The percentage of patients with missing data on Hugh-Jones grade was 25.1% for all patients with pneumonia, and this number was higher for patients with MRSA pneumonia (37.5%) before multiple imputation. Hospitalisation costs data were missing for 0.2% of all patients with pneumonia.

Table 3 shows the results of the multivariable logistic regression analysis with generalised estimating equations after multiple imputation for all-cause in-hospital mortality. MRSA pneumonia was significantly associated with higher mortality compared with non-MRSA pneumonia (adjusted odds ratio = 1.94; 95% confidence interval: 1.72–2.18; *p* < 0.001).

**Table 3:** Multivariable logistic regression analysis with generalised estimating equations accounting for clustering within matched pairs for all-cause in-hospital mortality

		Adjusted odds ratio	95% confidence interval	*p*-value
MRSA pneumonia		1.94	1.72–2.18	< 0.001
Sex (female)		0.59	0.52–0.67	< 0.001
Age (year)		1.01	1.00–1.01	0.001
Body mass index (kg/m^2^)	≤ 18.5	1.58	1.40–1.78	< 0.001
	18.5–24.9	Reference		
	25–29.9	0.70	0.54–0.91	0.007
	≥ 30	0.65	0.33–1.31	0.231
ADL score (Barthel Index)	85–100	Reference		
	60–80	1.05	0.80–1.37	0.724
	0–55	1.84	1.54–2.20	< 0.001
Hugh-Jones grade on admission	I	Reference		
	II	1.09	0.72–1.63	0.692
	III	1.59	1.07–2.38	0.023
	IV	2.22	1.57–3.14	< 0.001
	V	5.57	3.89–7.97	< 0.001
A-DROP score	0	Reference		
	1	1.46	0.94–2.28	0.093
	2	2.06	1.32–3.21	0.002
	3	3.05	1.94–4.78	< 0.001
	4	5.36	3.35–8.57	< 0.001
	5	11.8	7.13–19.6	< 0.001
CRP ≥ 20 mg/mL or infiltration covering at least two-thirds of one lung on chest radiography		1.38	1.17–1.64	< 0.001
Mechanical ventilation		1.84	1.48–2.29	< 0.001
at admission				
ICU admission		0.56	0.38–0.82	0.003
Haemodialysis		1.40	0.99–1.97	0.060
Emergency transport		1.01	0.91–1.13	0.837
COPD		0.77	0.65–0.90	0.002
Interstitial lung disease		1.84	1.41–2.40	< 0.001
Aspiration pneumonia		1.42	1.08–1.87	0.013
*Pseudomonas aeruginosa* pneumonia		0.95	0.68–1.32	0.743

MRSA, methicillin-resistant *Staphylococcus aureus*; ADL, activities of daily living; A-DROP, Age, Dehydration, Respiratory Failure, Orientation Disturbance and Blood Pressure; CRP, C-reactive protein; ICU, intensive care unit; COPD, chronic obstructive pulmonary disease

Higher mortality was significantly associated with lower BMI, lower Barthel Index, higher Hugh-Jones grade, higher A-DROP score, CRP ≥ 20 mg/dL or infiltration of least two-thirds of one lung, mechanical ventilation at admission, interstitial lung disease and aspiration pneumonia. The sensitivity analyses showed similar results to the main analyses (Additional file 1: Tables S1 and S2).

We performed the multiple linear regression analysis with generalised estimating equations after multiple imputation for hospitalisation costs. Hospitalisation costs were significantly higher for patients with MRSA pneumonia than for those with non-MRSA pneumonia (difference = United States Dollar (USD) 8502; 95% confidence interval: USD 7959–9045; *p* < 0.001). In the sensitivity analyses, hospitalisation costs were significantly higher for patients with MRSA pneumonia than for those with non-MRSA pneumonia (difference = USD 8457; 95% confidence interval: USD 7919–8996; *p* < 0.001).

## Discussion

Using a nationwide inpatient database in Japan, our study showed that mortality was higher in patients with MRSA pneumonia than in those with non-MRSA pneumonia. In addition, we showed that hospitalisation costs were higher for patients with MRSA pneumonia than for those with non-MRSA pneumonia.

In our study, in-hospital mortality among patients with MRSA pneumonia was 31.2%. Previous studies have reported MRSA pneumonia mortality to be around 30% [14, 15], which is comparable to our results.

Studies have shown conflicting results on the difference in mortality between patients with MRSA pneumonia and those with non-MRSA pneumonia. Some studies have shown higher mortality for patients with pneumonia caused by multidrug-resistant pathogens than for those with other types of pneumonia [2, 14], whereas other studies have found no significant differences [4, 16]. Several studies have shown high mortality in patients with MRSA bacteraemia [17–19], but few studies have focused on MRSA pneumonia. In the present study, we clearly demonstrated that mortality was twice as high in patients with MRSA pneumonia than in patients with non-MRSA pneumonia.

Additionally, previous studies have shown conflicting results on the difference in hospitalisation costs between MRSA and methicillin-sensitive *Staphylococcus aureus* pneumonia [20–22]. We confirmed that healthcare costs were higher for MRSA pneumonia than for non-MRSA pneumonia, including methicillin-sensitive *Staphylococcus aureus* pneumonia. Longer hospital stay may lead to higher hospitalisation costs in patients with MRSA pneumonia. Possible causes of the longer hospital stay are that patients with MRSA pneumonia are frailer and require longer duration of antibiotic therapy. The patients with MRSA pneumonia tended to have lower BMIs and lower activities of daily living scores. Although we adjusted for BMI and activities of daily living score, we were unable to fully evaluate frailty because the database lacked data on other components of the frailty definition, such as grip strength, exhaustion and slowness of walking [23].

Previous studies have shown several pulmonary comorbidities to be associated with higher mortality in patients with pneumonia, including interstitial lung disease [5, 24] and aspiration pneumonia [25]. The association between COPD and mortality remains uncertain in hospitalised adult patients with pneumonia [5, 26]. Our multivariable regression analysis included these comorbidities, finding no significant association between COPD and in-hospital mortality.

Limitations should be acknowledged. First, the database used for this study does not include bacterial culture or drug-susceptibility test results. We therefore combined an MRSA diagnosis and treatment for MRSA to identify patients with MRSA pneumonia. Second, the database does not include pulmonary function test results; thus, we were not able to account for the severity of pulmonary comorbidities. Finally, several factors, such as previous antibiotic use, were unmeasured, and we therefore could not eliminate confounding biases arising from these factors.

In conclusion, adjusted in-hospital mortality and hospitalisation costs were significantly higher for patients with MRSA pneumonia than for those with non-MRSA pneumonia in this matched-pair cohort study.

## Supplementary Information

**Additional file 1**. **Table S1**. Characteristics of patients with MRSA pneumonia and patients with non-MRSA pneumonia after 1:4 matching regarding with chronic heart failure, chronic liver diseases, sepsis, acute renal failure, leukopenia,immunosupression and stroke. Table S2; Sensitivity analyses adjusted comorbidities including chronic heart failure, chronicliver diseases, sepsis, acute renal failure, leukopenia,immunosupression and stroke for all-cause in-hospital mortality.

## Data Availability

The datasets generated and/or analysed during the current study are not publicly available.

## Abbreviations

MRSA: Methicillin-resistant *Staphylococcus* *aureus*
ICU: Intensive care unit
ICD-10: The International Classification of Disease and Related Health Problems, 10th Revision
A-DROP: Age-Dehydration-Respiratory failure-Orientation disturbance-blood Pressure
CRP: C-reactive protein
CURB-65: Confusion-Urea-Respiratory rate-Blood pressure-65
BMI: Body mass index
COPD: Chronic obstructive pulmonary disease
SD: Standard deviation
USD: United States Dollar
