# Supplementary material for: In-hospital mortality associated with community-acquired pneumonia due to methicillin-resistant Staphylococcus aureus: a matched-pair cohort study
Source: BMC Pulm Med. 2021 Nov 3;21:345. doi: 10.1186/s12890-021-01713-1 (PMC8564271; doi:10.1186/s12890-021-01713-1)
Supplement: Supplementary file 1 — Additional file 1. Table S1. Characteristics of patients with MRSA pneumonia and patients with non-MRSA pneumonia after 1:4 matching regarding with chronic heart failure, chronic liver diseases, sepsis, acute renal failure, leukopenia,immunosupression and stroke. Table S2; Sensitivity analyses adjusted comorbidities including chronic heart failure, chronicliver diseases, sepsis, acute renal failure, leukopenia,immunosupression and stroke for all-cause in-hospital mortality. [file 12890_2021_1713_MOESM1_ESM.docx]

**Additional file 1: Table S1** Characteristics of patients with MRSA pneumonia and patients with non-MRSA pneumonia after 1:4 matching regarding with chronic heart failure, chronic liver diseases, sepsis, acute renal failure, leukopenia, immunosuppression and stroke

|  | MRSA pneumonia | | Non-MRSA pneumonia | | *p*-value |
| --- | --- | --- | --- | --- | --- |
|  | *n* (%) | | *n* (%) | |  |
| Chronic heart failure |  |  |  |  | 0.025 |
| Yes | 647 | (20.9) | 2,350 | (19.1) |  |
| No | 2,455 | (79.1) | 9,970 | (80.9) |  |
| Chronic liver diseases |  |  |  |  | 0.519 |
| Yes | 7 | (0.23) | 21 | (0.17) |  |
| No | 3,095 | (99.8) | 12,299 | (99.8) |  |
| Sepsis |  |  |  |  | < 0.001 |
| Yes | 143 | (4.61) | 153 | (1.24) |  |
| No | 2,959 | (95.4) | 12,167 | (98.8) |  |
| Acute renal failure |  |  |  |  | 0.137 |
| Yes | 20 | (0.64) | 54 | (0.44) |  |
| No | 3,082 | (99.4) | 12,266 | (99.6) |  |
| Leukopenia |  |  |  |  | 0.665 |
| Yes | 10 | (0.32) | 34 | (0.28) |  |
| No | 3,092 | (99.7) | 12,286 | (99.7) |  |
| Immunosuppression |  |  |  |  | 0.568 |
| Yes | 3 | (0.10) | 17 | (0.14) |  |
| No | 3,099 | (99.9) | 12,303 | (99.9) |  |
| Stroke |  |  |  |  | < 0.001 |
| Yes | 420 | (13.5) | 1,229 | (9.98) |  |
| No | 2,682 | (86.5) | 11,091 | (90.0) |  |

MRSA: methicillin-resistant *Staphylococcus aureus*

**Additional file 1: Table S2** Sensitivity analyses adjusted comorbidities including chronic heart failure, chronic liver diseases, sepsis, acute renal failure, leukopenia, immunosuppression and stroke for all-cause in-hospital mortality

|  |  | Adjusted odds ratio | 95% confidence interval | *p*-value |
| --- | --- | --- | --- | --- |
| MRSA pneumonia |  | 1.95 | 1.73–2.20 | < 0.001 |
| Sex (female) |  | 0.58 | 0.51–0.66 | < 0.001 |
| Age (year) |  | 1.01 | 1.00–1.02 | 0.001 |
| Body mass index (kg/m^2^) | ≤ 18.5 | 1.60 | 1.43–1.80 | < 0.001 |
|  | 18.5–24.9 | Reference |  |  |
|  | 25–29.9 | 0.69 | 0.54–0.89 | 0.005 |
|  | ≥ 30 | 0.63 | 0.31–1.25 | 0.185 |
| ADL score (Barthel Index) | 85–100 | Reference |  |  |
|  | 60–80 | 1.05 | 0.79–1.39 | 0.726 |
|  | 0–55 | 1.87 | 1.56–2.24 | < 0.001 |
| Hugh-Jones grade on admission | I | Reference |  |  |
|  | II | 1.02 | 0.69–1.51 | 0.904 |
|  | III | 1.54 | 1.03–2.29 | 0.034 |
|  | IV | 2.17 | 1.51–3.10 | < 0.001 |
|  | V | 5.38 | 3.64–7.94 | < 0.001 |
| A-DROP score | 0 | Reference |  |  |
|  | 1 | 1.47 | 0.94–2.29 | 0.086 |
|  | 2 | 2.04 | 1.31–3.19 | 0.002 |
|  | 3 | 3.02 | 1.92–4.76 | < 0.001 |
|  | 4 | 5.25 | 3.29–8.39 | < 0.001 |
|  | 5 | 11.6 | 6.99–19.1 | < 0.001 |
| CRP ≥ 20 mg/mL or infiltration covering at least two-thirds of one lung on chest radiography |  | 1.36 | 1.16–1.59 | < 0.001 |
| Mechanical ventilation |  | 1.77 | 1.42-2.21 | < 0.001 |
| at admission |  |  |  |  |
| ICU admission |  | 0.53 | 0.36–0.78 | 0.001 |
| Haemodialysis |  | 1.36 | 0.95–1.93 | 0.089 |
| Emergency transport |  | 1.01 | 0.91–1.13 | 0.800 |
| COPD |  | 0.79 | 0.65–0.90 | 0.002 |
| Interstitial lung disease |  | 1.83 | 1.40–2.39 | < 0.001 |
| Aspiration pneumonia |  | 1.41 | 1.07–1.85 | 0.014 |
| *Pseudomonas aeruginosa* pneumonia |  | 0.96 | 0.68–1.34 | 0.792 |
| Chronic heart failure |  | 1.19 | 1.05–1.35 | 0.007 |
| Chronic liver diseases |  | 1.19 | 0.87–1.61 | 0.270 |
| Sepsis |  | 1.35 | 0.98–1.87 | 0.064 |
| Acute renal failure |  | 2.06 | 1.08–3.94 | 0.028 |
| Leukopenia |  | 0.88 | 0.32–2.47 | 0.812 |
| Immunosuppression |  | 1.46 | 0.19–11.2 | 0.717 |
| Stroke |  | 0.78 | 0.66–0.93 | 0.004 |

MRSA: methicillin-resistant *Staphylococcus aureus*; ADL: activities of daily living; A-DROP: Age, Dehydration, Respiratory Failure, Orientation Disturbance and Blood Pressure; CRP: C-reactive protein; ICU: intensive care unit; COPD: chronic obstructive pulmonary disease
